# Multimodal Imaging of Lamellar Macular Holes

**DOI:** 10.1155/2021/8820444

**Published:** 2021-01-30

**Authors:** Roberto dell'Omo, Mariaelena Filippelli, Serena De Turris, Andrea Govetto, Pasquale Napolitano, Ermanno dell'Omo, Ciro Costagliola

**Affiliations:** ^1^Department of Medicine and Health Sciences “Vincenzo Tiberio”, University of Molise, Via Francesco De Sanctis 1, Campobasso 86100, Italy; ^2^Eye Clinic, Polytechnic University of Marche, Via Conca 71, Ancona 60121, Italy; ^3^Vitreoretinal Division, Bristol Eye Hospital, Lower Maudlin St., BS1 2LX, Bristol, UK; ^4^Eye Clinic, Casa di Cura “Villa Maria”, Viale Principe di Piemonte 4, Campobasso 86100, Italy

## Abstract

Evolution of imaging techniques has renewed interest in the diagnosis of lamellar macular hole (LMH) and greatly implemented the possibilities of gaining more detailed insights into its pathogenesis. Among noninvasive techniques, optical coherence tomography (OCT) is considered the primary examination modality to study LMHs, given its ability to image foveal structure and its widespread availability. OCT also allows to resolve the epiretinal materials associated with LMH, i.e., tractional epiretinal membranes (ERMs) and epiretinal proliferation (EP). En face OCT reconstructions are useful to confirm the foveal abnormalities shown by the eyes with LMH, whereas OCT angiography may reveal alterations of the size and shape of the foveal avascular zone and alterations of the density of the superficial and deep vascular plexuses. On slit-lamp biomicroscopy or fundus camera examination, LMH appears as a round or oval, reddish lesion at the center of the macula, slightly darker than the surrounding retina. The associated tractional ERM, causing wrinkling and glistening of the retinal surface, is usually readily appreciable, whereas EP is hardly apparent on biomicroscopy or fundus photography since the retina surface appears smooth. When imaged with blue fundus autofluorescence (B-FAF) imaging, LMHs are characterized by an increased autofluorescent signal, the intensity of which does not correlate with the thickness of the residual outer retinal tissue. Green reflectance and blue reflectance (BR) images clearly show the increased reflection and wrinkling of the retinal surface caused by tractional ERM associated with LMH. BR and multicolor imaging enable the visualization of EP associated with LMH in the form of a sharply demarcated dark area and in the form of a yellowish area surrounding the hole, respectively. Scarce data regarding invasive imaging techniques, such as fluorescein angiography, for the study of LMH are available in the literature. The aim of this review is to evaluate the contribution that each imaging modality can provide to study the morphologic characteristics of LMH.

## 1. Background

The term lamellar macular hole (LMH) was introduced in 1975 by Gass [1], who identified, by slit-lamp biomicroscopy, an oval reddish macular lesion resulting from cystoid macular edema, secondary to the rupture of the roof of a foveal cyst. In the following years, the term LMH has been used to refer to foveal alterations with certain characteristic on biomicroscopic examination, independent of the pathogenesis (idiopathic or secondary to other pathologies).

Evolution of imaging techniques and especially the advent of widespread use of optical coherence tomography (OCT) has renewed interest in the diagnosis of LMH and greatly implemented the possibilities of gaining more detailed insights into its pathogenesis.

Preliminary studies based on time-domain OCT suggested that LMH could be the consequence of an aborted process of full-thickness macular hole formation, leading to avulsion of part of the inner fovea because of vitreofoveal traction [2, 3]. Other studies proposed that anteroposterior and tangential forces exerting centripetal or centrifugal traction on the fovea might be involved in the pathogenesis of LMH [4, 5]. However, more recent spectral-domain OCT studies challenged these assumptions suggesting that true LMH might be the result of remodelling of the foveal tissue occurring in absence of overt epiretinal tractional forces [6–10]. True LMHs are often associated with a tissue of intermediate reflectivity, usually thicker than standard, tractional epiretinal membranes (ERMs), observed on the retinal surface. This tissue, originally described by Witkin et al. [4] as “thickened ERM,” subsequently renamed “lamellar hole-associated epiretinal proliferation” [8] is nowadays referred to as epiretinal proliferation (EP) [10] since it is not exclusive to LMH but can be found also in a wide spectrum of retinal diseases, including full-thickness macular holes, posterior uveitis, macular pucker, age-related macular degeneration, diabetic retinopathy, refractory macular edema, vein occlusion, and high myopia [11].

Histopathologic studies have evidenced significant differences in the composition of tractional ERM versus EP. [6, 12, 13]. Specifically, myofibroblasts dominate in highly reflective membranes, whereas membranes of medium reflectivity consist primarily of fibroblasts and hyalocytes.

In order to propose a clear definition of LMH based on new retinal imaging and to differentiate LMH from other similar but distinguishable entities like schisis of the fovea associated with ERM and pseudohole, an international panel of vitreoretinal experts has recently proposed new OCT-based criteria for the diagnosis of these three entities. According to the panel, the features characterizing LMH are the presence of irregular foveal contour, foveal cavity with undermined edges, and presence of pseudooperculum and/or thinning of the fovea. Associated pathological changes can include EP, foveal bump, and ellipsoid line disruption. Such a definition is similar to what was previously considered as a ‘true' or degenerative LMH [14, 15].

Conversely, schisis of the fovea associated with ERM has been named ERM foveoschisis and features the presence of contractile ERM, foveoschisis at the level of Henle fiber layer (HFL) and optionally microcystoid spaces in the inner nuclear layer (INL), and retinal thickening and wrinkling. In the literature, this entity has been previously referred to as “tractional” LMH and/or pseudohole with lamellar cleavage of its edges [14, 15].

Finally, macular pseudohole (PSH) features a foveal center sparing ERM, retinal thickening with verticalised or steepened foveal profile, and optionally presence of microcystoid spaces in the INL and near-normal central foveal thickness. Such a definition is similar to that previously proposed by the International Vitreomacular Traction Study Group [16].

One main aspect differentiating LMH from ERM foveoschisis and PSH resides in the assumption that only LMH is associated with loss of tissue. As specified earlier, presumed signs of retinal cell loss on OCT in presence of LMH are the undermined edges, foveal thinning, and a posterior vitreous detachment associated with pseudooperculum. However, OCT imaging may not be fully reliable in distinguishing loss of tissue.

It was proposed that blue fundus autofluorescence (B-FAF), another imaging modality, could overcome some limits of OCT and discriminate loss of tissue by showing an increased autofluorescent signal at the fovea [17]. Nevertheless, ERM foveoschisis, LMH, and PSH all feature an increased autofluorescent signal at the fovea. Furthermore, there is uncertainty if the increased autofluorescence associated with LMH and PSH represents an actual loss of foveal tissue or a centrifugal displacement of neurosensory tissue containing macular pigment or a combination of both [17–19].

More recently, the potentialities of multicolor imaging (MCI) in studying LMH and in particular the EP associated with LMH have been investigated. Nevertheless, like B-FAF, MCI does not provide clues regarding the loss of tissue.

Thus, despite advancements in imaging technology, at present only histological studies appear able to definitely confirm the presence of retinal tissue loss associated with LMH.

The aim of the present paper is to give an overview about the imaging modalities used to study LMH and the contribution that each of them can give in better understanding the pathogenesis of this fascinating vitreomacular disease.

## 2. Methods

To identify and select the relevant articles regarding retinal imaging features in eyes with LMH, a research was performed on PubMed (https://www. ncbi. nlm. nih. gov/ pubmed/) using the following terms (or combination of terms): “lamellar macular hole” or “LMH” or “epiretinal membrane” or “ERM” or “lamellar hole-associated epiretinal proliferation” or “LHEP” or “epiretinal proliferation” or “EP” or “foveoschisis”, last accessed 17 August 2020.

During the period under review, patients with a LMH and an ERM foveoschisis were classified as “degenerative LMH,” or “tractional LMH” or “lamellar hole”.

**Figure 1:**
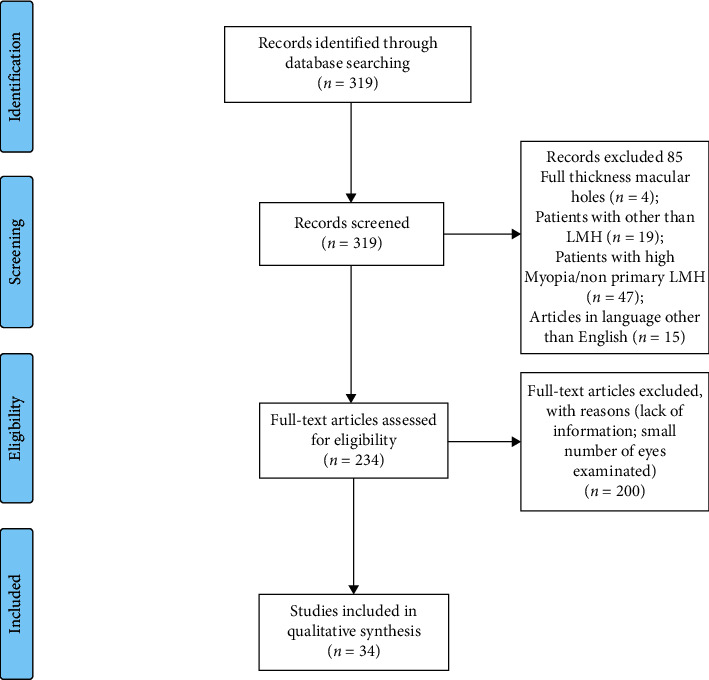
Flow diagram showing the methodology followed to review the literature and select the papers of interest.

All references of the included articles were also screened to guarantee no omission of literature. For study selection, the inclusion criteria were (1) accurate description of LMH features using imaging techniques and (2) articles written in English. The selection was then reviewed by the authors and a final list of 42 papers agreed (Figure 1) to be used as the basis for the review. Of these, 34 directly regarded LMH, whereas the remaining 8 were considered for the differential diagnosis of LMH with similar conditions or for the description of multimodal imaging techniques.

## 3. Results

### 3.1. Slit-Lamp Biomicroscopy and Fundus Camera Photography

**Figure 2:**
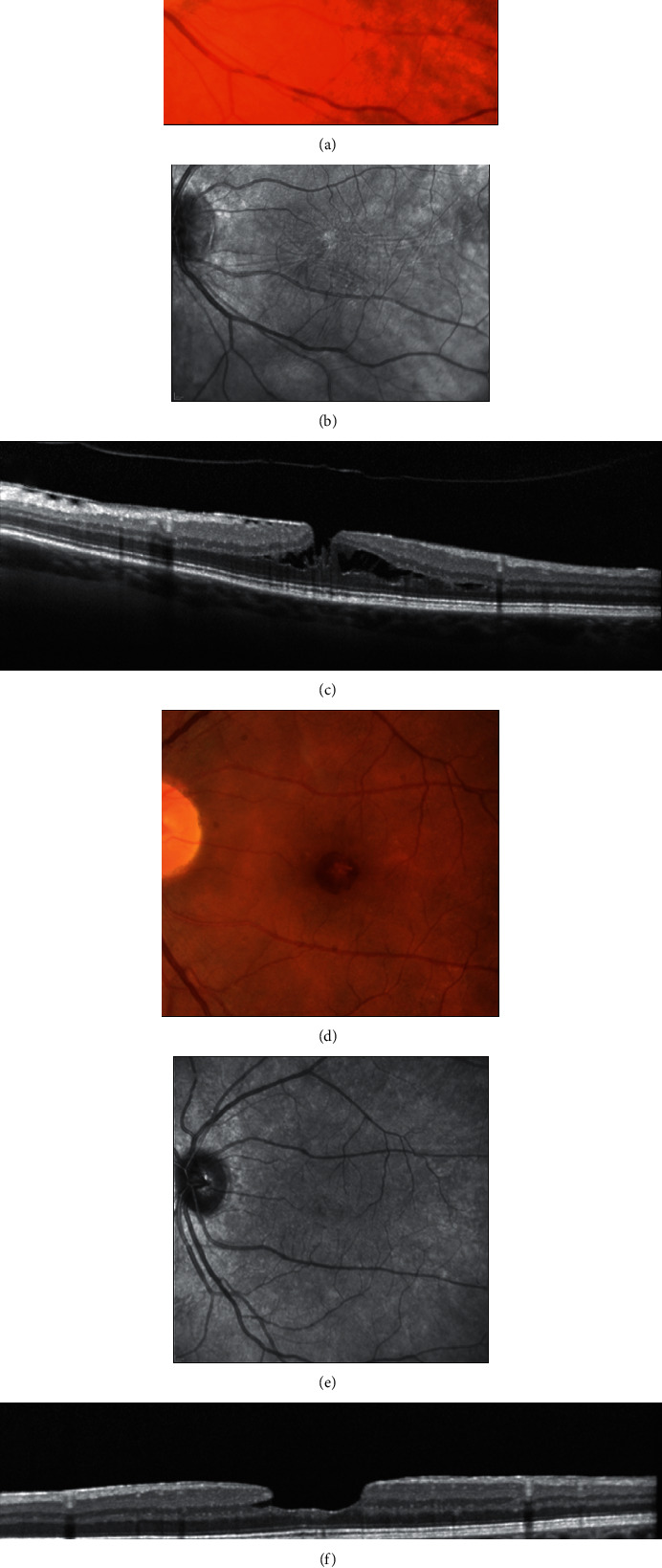
(a–c) Fundus camera (FC) color photograph, infrared (IR) image, and optical coherence tomography (OCT) of epiretinal membrane (ERM) foveoschisis. (a) On FC there is an oval reddish lesion at the fovea, (b) whereas on IR image, wrinkling of the retinal surface is appreciated; (c) on OCT there is an ERM over the inner limiting membrane (ILM) with the presence of hyporeflective spaces between the ERM and the ILM and a foveoschisis at the level of Henle fiber layer. (d–f) FC color photograph, IR image, and OCT of lamellar macular hole (LMH). (d) On FC a round reddish lesion at the fovea is noted; (e) on IR image the retinal surface appears smooth; (f) on OCT irregular foveal contour, foveal cavity with undermined edges, thinning of the fovea, foveal bump, and ellipsoid line disruption are present.

On slit-lamp biomicroscopy or fundus camera (FC) examination (Figure 2), LMH appears as a round or oval, reddish lesion at the center of the macula, slightly darker than the surrounding retina [1]. The associated EP, if present, is hardly apparent on biomicroscopy or fundus photography since the retina surface appears smooth [8]. A central reddish lesion is also visible in ERM foveoschisis. The associated tractional ERM, causing wrinkling and glistening of the retinal surface, is usually readily appreciable on fundus examination.

In the time-domain OCT era, Haouchine et al. [3] showed that only 28% of LMH cases diagnosed with OCT were recognized as LMH on fundus examination. Likewise, Witkin et al. [4] reported that only 37% of LMHs diagnosed using an ultrahigh resolution OCT were detected clinically on fundus examination. It must be noted that, in these series, true LMH and ERM foveoschisis were both grouped under the unique definition of LMH.

However, these data show the limits of slit-lamp biomicroscopy and fundus photography for the correct diagnosis of LMH and for the differential diagnosis with similar-looking macular pathologies.

### 3.2. Optical Coherence Tomography

**Figure 3:**
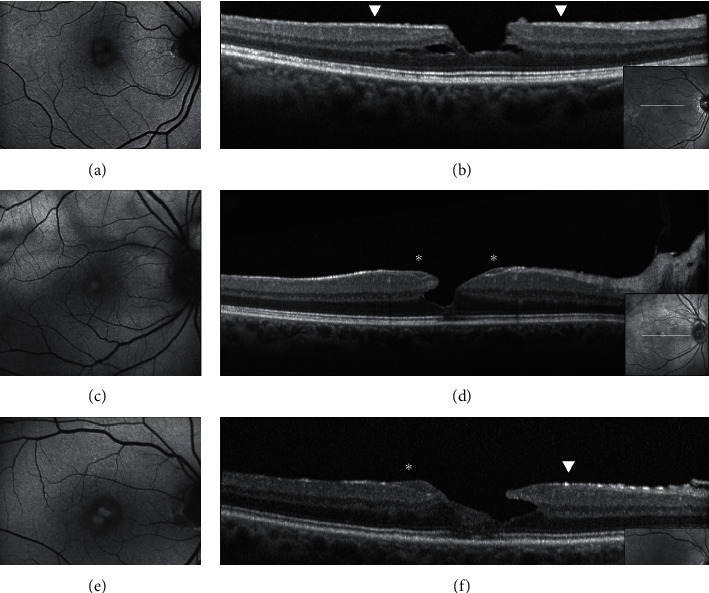
Blue fundus autofluorescence (B-FAF) (a, c, e) and optical coherence tomography images (OCT) (b, d, f) in eyes with epiretinal membrane (ERM) foveoschisis and lamellar macular hole (LMH). On B-FAF images, areas of increased autofluorescence at the center of the fovea can be present in both conditions. On OCT images, ERM foveoschisis is, by definition, associated with a tractional epiretinal membrane (arrowheads, b), whereas LMH may be associated with epiretinal proliferation (EP, d, asterisks) or with concomitant ERM and EP (f, arrowhead and asterisk, respectively). Intraretinal schisis and usually intact external limiting membrane (ELM) and ellipsoid zone (EZ) are noted in presence of ERM foveoschisis (b) whereas undermined edges and disrupted ELM and EZ are noted in presence of LMH. The horizontal white arrows on the infrared image (small squares within the B-FAF images) indicate the location of the corresponding OCT scans.

Using time-domain OCT, Haouchine et al. [3] first defined the criteria for the OCT-based diagnosis of LMH. Later, Witkin et al. [4] refined the classification using ultrahigh-resolution OCT. They proposed 4 basic criteria: (1) an irregular foveal contour; (2) a break in the inner fovea; (3) a dehiscence of the inner foveal retina from the outer retina occurring either between outer plexiform layer (OPL) and outer nuclear layer or only within the ONL; and (4) an absence of a full-thickness foveal defect with intact foveal photoreceptors. Of note, Witkin and associates reported two distinct appearances of epiretinal material associated with LMH: one was constituted by a thin highly reflective line immediately anterior to and separate from the retinal nerve fiber layer (RNFL); the other was a moderately reflective material filling the space between the outer border of the ERM and RNFL and it was named “unusual thick membrane” (Figures 2 and 3). A few years later, Parolini et al. [6] renamed these “thick membranes” as “dense membranes” and showed irregularities and disruption of the outer retinal bands (ORB) at the fovea (i.e., external limiting membrane and ellipsoid and interdigitation zone) in association with them, thus challenging the validity of criterion 4 (intact photoreceptors) of the classification proposed by Witkin and associates. On the basis of OCT and histopathologic analysis, Parolini et al. [6] proposed to make a distinction between tractional membranes (thin and hyperreflective on OCT and with predominance of *α*-smooth muscle actin [SMA]-positive cells on histology analysis) and dense membranes (thick on OCT and with predominance of collagen fibrils on histology analysis) associated with LMH. For these reasons and based on different outcomes after surgery, Gaudric et al. [14] subsequently proposed that LMH with tractional membranes should be regarded as a subcategory of PSH in which a lamellar dissection caused by the separation of the inner and outer retinal layers had occurred. Gaudric et al. suggested that the name “macular pseudoholes with lamellar cleavage of their edges” (instead of LMH) would be more appropriate to define these foveal lesions. In 2014, Pang et al. [8] renamed the formerly described “thick” or “dense” epiretinal material as “lamellar hole-associated epiretinal proliferation” (LHEP). They found LHEP, a material of intermediate reflectivity and of variable thickness in 30.5% of the eyes with LMH of their retrospective series. Reportedly, 97% of these eyes with LHEP had disruption of the ellipsoid zone and 88% had visible connecting tissue from the base of the LMH to LHEP, this feature suggesting that LHEP may originate from within the inner retinal defect. Of interest, despite they noticed that LHEP did not induce tractional effects such as distortion or edema of the underlying normal retinal tissue, splitting of the retina in the region of Henle fiber layer (HFL) was reported in 98% of eyes with LHEP. On the basis of these imaging observations, Pang et al. reiterated the hypothesis originally proposed by Parolini and Bottoni et al. [6, 7] that the two types of LMH associated with different types of epiretinal membranes may have different pathogenetic origin. In 2016, taking into consideration the characteristics of the epiretinal material associated with LMH and other specific features on OCT imaging, Govetto et al. [15] proposed to classify LMH in 2 types: degenerative and tractional LMH. The degenerative type was characterized by the presence of EP, ratio between inner and outer diameter of the hole of more than 1 : 2, presence of a foveal bump, a round-edged intraretinal cavitation, and, in the large majority of the cases, a disrupted ellipsoid zone. The tractional type was characterized by the presence of a tractional membrane, a ratio between inner and outer diameter of the hole generally less than 1 : 2, intact ellipsoid layer, and a sharp-edged schisis-like appearance between outer plexiform and outer nuclear layers. In the series by Govetto et al., 10.78% of the eyes examined shared common features of both degenerative and tractional LMH. Other series confirmed the occurrence of these “mixed-cases” and reported the concomitant presence of EP and tractional ERM in a substantial higher number of cases [17–19]. Further studies reported that the presence of EP resulted frequently associated with peculiar morphologic and functional features of LMH. For instance, a worse BCVA, a thinner CFT, a cavitated appearance of the retina, and the disruption of the outer retinal bands were typically associated with EP [6–9, 14, 17, 18–20]. On the other hand, other OCT features were similar in lamellar macular defects associated or not with EP. For example, measurements of the horizontal and vertical diameters of the holes at the level of the OPL and their stability over time appeared rather similar in cases with and without EP [19].

In 2020, Hubschman [10] and a panel of vitreoretinal experts proposed a new OCT-based definition of LMH based on three mandatory and three optional criteria. Among the mandatory criteria (irregular foveal contour, foveal cavity with undermined edges, and other signs evoking a loss of foveal tissue), the concept of a foveal cavity with undermined edges is considered as a key feature of LMH because it is regarded as highly suggestive of retinal cell loss. A cavity with undermined edges is characterized by an angle between the surface of the retina and the edge of the hole lower than 90°; such morphology must be present in at least two B-scans separated 242 *μ*m apart to make the diagnosis valid. In this classification, the occurrence of EP, ellipsoid zone disruption, and foveal bump is considered optional for the diagnosis because it is not always present in association with LMH. The lesions showing the above reported mandatory signs may be referred to as “primary” and “nonprimary” LMH, depending on the aetiology, respectively, idiopathic or secondary to known pathologies. Such a definition is similar to what was previously considered as a “true” or degenerative LMH [14, 15].

**Figure 4:**
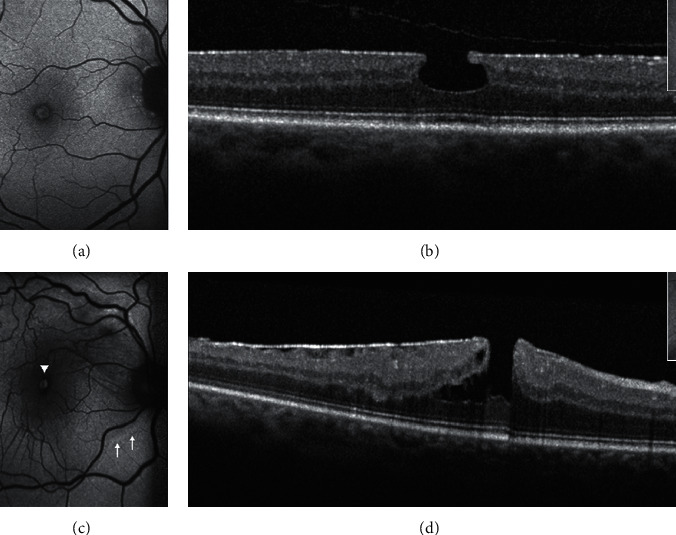
Blue fundus autofluorescence (B-FAF) and optical coherence tomography images (OCT) in an eye with standard macular pseudohole (PSH) and in an eye with PSH with lamellar cleavage of its edges according to Gaudric et al. [14]. B-FAF (a, c): an increased autofluorescent signal is present at the fovea in both cases. On OCT (b), standard PSH is characterized by foveal centre sparing epiretinal membrane, retinal thickening, verticalised/steepened foveal profile, and near normal central foveal thickness. PSH with lamellar cleavage of its edges (d) shows in addition an intraretinal schisis. and has been reclassified as epiretinal membrane foveoschisis by Hubschman and coworkers [10].

Conversely, the cases previously referred to in the literature as “tractional” LMH and “macular pseudohole with stretched edges” have been renamed by the panel as “ERM foveoschisis” **(**Figure 4**)**. The two mandatory criteria for the diagnosis of ERM foveoschisis are the presence of a contractile ERM and foveoschisis at the level of HFL; optional criteria are the presence of microcystoid spaces in the INL, retinal thickening, and retinal wrinkling.

**Figure 5:**
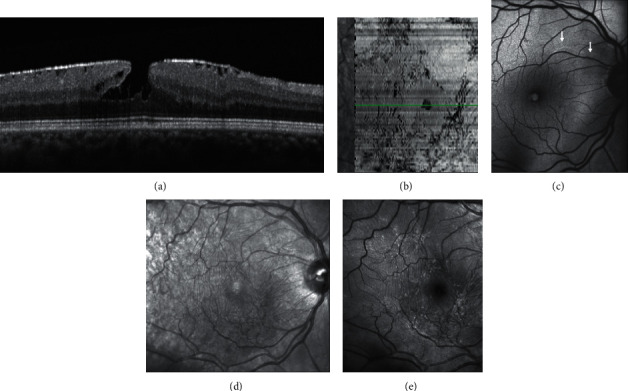
Multimodal imaging of epiretinal membrane (ERM) foveoschisis. (a) Structural optical coherence tomography (OCT) shows the hyperreflective line corresponding to the ERM on the retinal surface and the intraretinal schisis at the level of the Henle fiber layer. (b) On en face OCT (segmentation at the level of the vitreoretinal interface) signs of traction like folds and retinal wrinkling are visible in the macular area. The horizontal green line indicates the location of the corresponding structural OCT scan. (c) Blue fundus autofluorescence shows an area of increased signal at the fovea and retinal vessel printings at the superonasal aspect of the macula (arrows). Superficial wrinkling of the inner retina is notable on the infrared image (d) but is better visualized on the green reflectance image (e) where several foci of traction are also evident.

**Figure 6:**
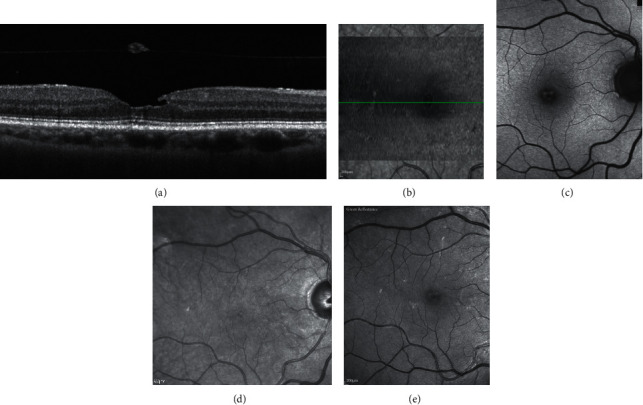
Multimodal imaging of lamellar macular hole. (a) Structural optical coherence tomography (OCT) shows irregular foveal contour, foveal cavity with undermined edges, posterior vitreous detachment with pseudooperculum, and thinning of the fovea at its center. (b) On en face OCT (segmentation at the level of the vitreoretinal interface), no signs of traction like folds and retinal wrinkling are visible in the macular area. Blue-fundus autofluorescence (c) shows an area of increased signal at the fovea partially masked by the pseudo-operculum. On infrared (d) and on green-reflectance (e) images, the retinal surface appears smooth.

In contradistinction to ERM foveoschisis characterized by the presence of contractile ERM by definition and foveoschisis at the level of HFL, mechanical tangential traction does not seem to be relevant in LMH (Figures 2, 3, 5, and 6).

### 3.3. En Face OCT

En face OCT images are coronal-view images generated after computerized flattening along a specific retinal layer boundary that allow layer-by-layer analysis of the retinal tissue and may overcome some limitations of OCT cross-sectional images that may not provide detailed evaluation of traction, including traction strength and direction [21].

Gaudric et al. [14] noted that, in eyes with tractional ERM causing splitting of the retinal tissue, en face OCT images focused on the ERM showed several eccentric epicenters of contraction while images focused on the inner retina showed intraretinal folds induced by the ERM contraction. A rather similar aspect was observed in eyes with PSH in which en face OCT image focused on the surface of the internal limiting membrane (ILM slab) showed the radial folds of the ERM and of the inner limiting membrane converging toward the fovea, i.e., the epicenter of the membrane contraction in PSH. As specified above, on the basis of cross-sectional and en face OCT images, the authors proposed that macular pseudoholes with lamellar cleavage of their edge must be considered pseudoholes **(**Figure 4). In fact, en face OCT focused on the retinal surface and on the inner retina in case of true LMH shows a smooth retinal surface without any retinal folds [14] (Figures 5 and 6**)**.

Using en face OCT, Clamp et al. [21] measured the area of intraretinal splitting in 42 eyes with lamellar macular defects. All the 42 eyes included in the study exhibited an area of intraretinal schisis according to the definition proposed by Witkin et al. [4] and only 7 did not present with an ERM. Thus, it is likely that the majority of “LMHs” described in this series were ERM foveoschisis rather than true LMH according to the most recent classification by Hubschman and associates. Clamp et al. [21] found that the eyes with ellipsoid zone disruption had significantly greater mean intraretinal schisis area and worse mean visual acuity than eyes without ellipsoid disruption. They also found a strong relationship between horizontal linear schisis diameter and the area of intraretinal schisis but the relationship was not linear, as would be expected if all of the foveal lesions were circular. Instead, some LMHs demonstrated stellate, ovoid, or semicircular pattern with the greatest linear diameter along the vertical or oblique axis. The strands of retinal tissue within the intraretinal split observed on en face images were interpreted as Müller cell and photoreceptor cell processes as originally proposed by Witkin et al. [4]. However, this pattern is not pathognomonic for LMH because it is seen in other vitreoretinal interface disorders including vitreomacular traction syndrome, full-thickness macular hole (FTMH), and myopic schisis. In 98% of the cases, the intraretinal splitting occurred within the border between HFL and the synaptic component of the outer plexiform layer.

Using en face OCT, Hirano et al. [22] identified 3 groups of LMHs, all characterized by the presence of an ERM: the first group lacked retinal folds and parafoveal epicenter of constriction (PEC) in the ERM (PEC-ERM) sign of a localized strong contraction in the parafovea; ellipsoid zone disruption and LHEP were seen in 69% and 81% of this group, respectively. The other two groups presented retinal folds associated with retinal cleavage and retinal folds associated with retinal cleavage and PEC-ERM, respectively. As reported by the authors, 81% of the eyes in the first group could be classified as “degenerative LMH” according to Govetto et al. [15], whereas the eyes in the second and third group were classifiable as tractional LMH in 95% and 100% of the cases, respectively. By contrast, PSH showed retinal folds but lacked PEC-ERM and retinal cleavage. Based on these results, the authors reiterated Gaudric's hypothesis that MPHs with stretched edges are part of the spectrum of MPH and are induced by ERM contraction. Nevertheless, on the basis of the current imaging technology, they acknowledged the difficulty of supporting the assumption of Takahashi [2] and Pang [13] who suggested that the loss of inner foveal tissue is a key characteristic of true LMH.

**Figure 7:**
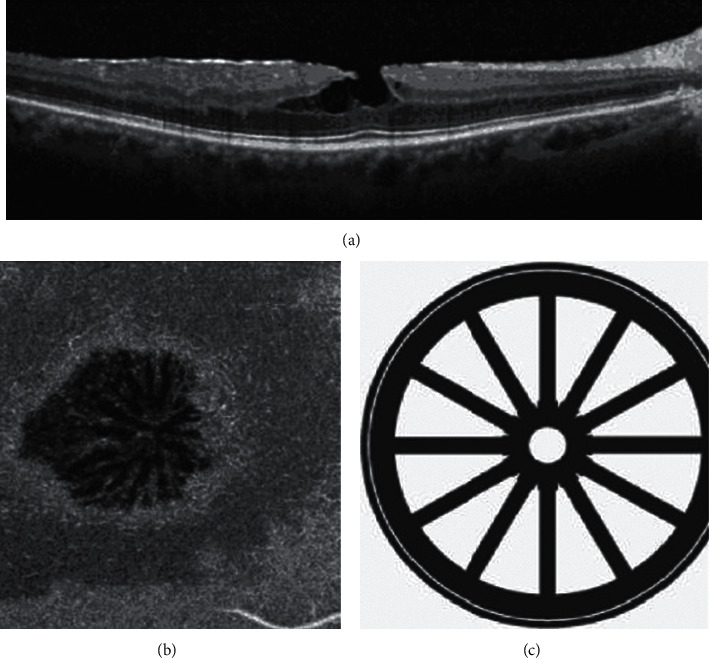
Structural and en face optical coherence tomography (OCT) of epiretinal membrane (ERM) foveoschisis. A structural OCT illustrates an ERM foveoschisis, with a sharp split at the level of the outer nuclear-Henle fiber layers complex. (a) Tractional ERM is visible. (b) The en face OCT segmented at the level of the outer nuclear-Henle fiber layers complex illustrates hyporeflective intraretinal cystoid spaces disposed in a radial pattern centered into the fovea. Such disposition may recall a “spoke-wheel” shape as shown in the drawing (c).

In a recent multimodal imaging analysis, Govetto et al. [23] showed that ERM foveoschisis presents with a characteristic radial “spoke-wheel” appearance on en face OCT imaging (segmented at the level of the outer nuclear layer-HFL complex) and this appearance would be consistent with the disposition of parafoveal Müller cells (Figure 7). Comparing en face OCT and fluorescein angiography findings, the authors speculated that the intraretinal splitting characteristic of ERM foveoschisis may be considered a subtype of macular edema in which intraretinal spaces are created by mechanical displacement of cells rather than disruption of the inner and/or outer retinal barriers. This assumption fits well with previously published studies on the biomechanics of the parafoveal Müller cells [24].

### 3.4. Blue Fundus Autofluorescence

Blue fundus autofluorescence (B-FAF) imaging is a modality that relies on the fluorescence generated by the bisretinoids of lipofuscin in retinal pigment epithelial (RPE) cells and is influenced by absorbent or autofluorescent materials anterior to the RPE monolayer [25, 26]. The autofluorescent signal may be recorded by a confocal scanning laser ophthalmoscope (cSLO) or by a fundus camera. Confocal scanning laser ophthalmoscopes have a confocal capability by which only conjugate points on the fundus are imaged whereas points not lying on the conjugate planes are rejected. Commercially available cSLO use an excitation wavelength of 488 nm generated by an argon or solid-state laser and a wide band-pass barrier filter with a short wavelength cutoff inserted in front of the detector set at around 500 nm. By contrast, fundus camera system uses an excitation filter from 535 to 580 nm and a barrier filter from 615 to 715 nm. In eyes without disease-related abnormalities, B-FAF imaging of the macula shows reduced signal at the fovea (because of absorption of the blue light by the macular pigment) and a distinct increase at about the foveal margin, followed by a further gradual increment toward the outer macula.

When imaged with B-FAF imaging, LMHs and ERM foveoschisis are characterized by an increased autofluorescent signal, the intensity of which does not correlate with the thickness of the residual outer retinal tissue [17]. Interestingly, lesions classified as PSH on the basis of OCT may have an appearance similar to LMH and ERM foveoschisis on B-FAF imaging [17].

The significance of this area of increased B-FAF signal is not clear. This feature might represent an actual loss of foveal tissue or a mere centrifugal displacement of neurosensory tissue containing macular pigment or both [17, 19]. At present, B-FAF cannot answer this question.

In a study by dell'Omo et al., the increased autofluorescent signal at the fovea appeared to be similar in size in the presence of lamellar holes associated with tractional ERM or EP [19]. In fact, diameters of the holes measured on B-FAF images did not differ between eyes with and without EP at baseline and did not change significantly during the follow-up period [19].

Thus, LMHs, foveoschisis ERM, and PSH, although with different features based on OCT, may appear to be indistinguishable based on FAF imaging (Figures 3 and 4). Interestingly, the size of the area of increased autofluorescence may decrease after surgery both in eyes with ERM foveoschisis and in eyes with true LMH associated with EP. This seems to suggest that, at least in part, the increased autofluorescent signal observed preoperatively in eyes with LMH and ERM foveoschisis originates from displaced rather than lacking retinal tissue. Another application of B-FAF in eyes with ERM foveoschisis is its capacity of showing the presence of retinal vessel printings (RVPs), which are a useful sign for indirectly evaluating the tangential traction related to ERM [27, 28] (Figures 4 and 5).

In a retrospective series [19] of 84 eyes with lamellar defect at the fovea, RVPs were detected in none of the 11 eyes with associated EP, in 16.3% of the eyes with tractional ERM, and in 7.3% of eyes with the coexistence of EP and ERM. These results appear to confirm the scarce contractile characteristics of EP.

**Figure 8:**
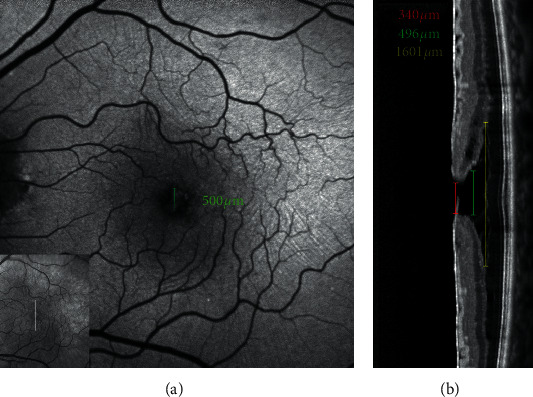
Blue fundus autofluorescence (a) and optical coherence tomography (OCT, b)-based measurements in an eye with epiretinal membrane foveoschisis. The horizontal white arrow on the infrared image (small square within the B-FAF image) indicates the location of the corresponding OCT scans; the green caliper on the B-FAF image indicates where the diameter of the increased area of autofluorescence was measured. The measurements on OCT image are taken at the level of the inner limiting membrane (red line), Henle fiber layer (green line), and schisis (yellow line) level. Note the similarity between the diameter of the area of increased autofluorescence measured from B-FAF image and the diameter measured at the level of the Henle fiber layer from OCT image.

**Figure 9:**
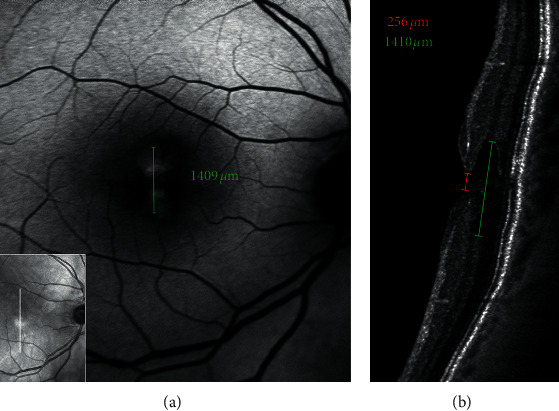
Blue fundus autofluorescence (a) and optical coherence tomography (OCT, b)-based measurements in an eye with lamellar macular hole. The horizontal white arrow on the infrared image (small square within the B-FAF image) indicates the location of the corresponding OCT scans; the green caliper on the B-FAF image indicates where the diameter of the increased area of autofluorescence was measured. The measurements on OCT image are taken at the level of the inner limiting membrane (red line) and Henle fiber layer (green line). Note the similarity between the diameter of the area of increased autofluorescence measured from B-FAF image and the diameter measured at the level of the Henle fiber layer from OCT image.

In an observational three-center study in which patients with lamellar defect were examined with B-FAF and SD-OCT according to prespecified imaging protocols, dell'Omo et al. [29] found that, independently from the associated epiretinal material (tractional ERM or EP), a strong correlation exists between the diameters of the holes measured from B-FAF images and those measured at the OPL level from OCT images (Figures 8 and 9). Conversely, no correlation was found between the length of disrupted EZ and B-FAF diameter. This is important because areas with disrupted EZ on OCT images (indirectly suggesting loss or rarefaction of photoreceptors) may potentially show increased B-FAF levels relative to surrounding areas with healthy photoreceptors. In fact, unbleached photoreceptor pigment has a similar, although lesser, effect on the appearance of B-FAF as macular pigment, as it absorbs and therefore attenuates the excitation light available to elicit autofluorescence at the level of the RPE [30].

These findings suggest that the loss or displacement of retinal tissue within the OPL layer might be the main culprit of the increased B-FAF signal observed in eyes affected by macular lamellar defects associated with either tractional ERM or EP.

**Figure 10:**
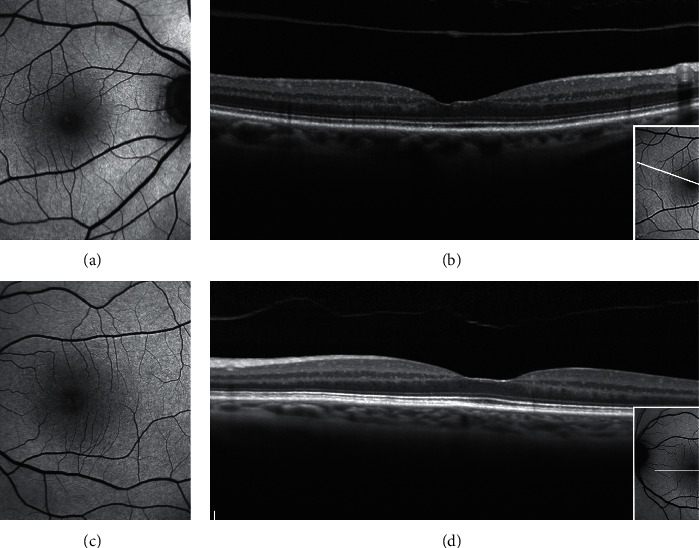
B-FAF and OCT imaging of Foveal Abnormality associated with epiretinal Tissue of medium reflectivity and Increased blue-light fundus Autofluorescence Signal (FATIAS). In the step type, the B-FAF image shows an area of increased autofluorescent signal (a), and, in the OCT image, there is an asymmetric contour of the foveal pit with one side more elevated than the other (b). The white lines on the B-FAF images in the small squares indicate the OCT scan level. In the rail type, the B-FAF image shows an increased autofluorescent signal at the fovea (c), and the OCT profile is characterized by a shallow foveal pit and a rail of tissue of medium reflectivity that is thicker in the central part and thinner at the edges of the foveal pit and that is similar to epiretinal proliferation (d).

Recently, dell'Omo et al. [31] described a distinct vitreomacular interface disorder termed foveal abnormality associated with epiretinal tissue of medium reflectivity and increased blue light fundus autofluorescence signal (FATIAS) **(**Figure 10**)**. Distinguishing features of FATIAS are an abnormal foveal contour either in the form of a step or in the form of a shallow foveal pit and reduced foveal thickness on SD-OCT imaging; the presence of a tissue of medium reflectivity on the innermost portion of the foveal pit; the absence of overt ERM or EP; the absence of intraretinal cysts or splitting/cavitation between the inner and outer retinal layers; the integrity of the outer retinal bands; an increased B-FAF signal at the fovea; good BCVA. It is possible that some cases of FATIAS may represent LMH in an early stage, although the retrospective analysis by dell'Omo et al. showed that none of the cases worsened morphologically or functionally over years-long follow-up.

### 3.5. Blue Reflectance, Green Reflectance, Infrared, and Multicolor Imaging

The characteristics of the epiretinal materials associated with LMH and ERM foveoschisis are of great relevance since a tractional ERM or EP can be found in more than 80% of the eyes with LMH and, a tractional ERM is found, by definition, in 100% of the eyes with ERM foveoschisis [10].

Traditional color fundus photography taken with FC uses a flash of white light to illuminate the retina and image quality may suffer because of light scattering, a broad depth of focus, poor pupil dilation, or media opacities. Alternatively, cSLO uses scanning laser to produce en face grayscale images that may hold several advantages over FC including higher spatial resolution, narrow depth of focus, and better penetration through a small pupil or media opacities [32].

The Spectralis (Heidelberg Engineering, Heidelberg, Germany) uses a cSLO to capture three simultaneous reflectance images in three different laser wavelengths: (1) blue reflectance (BR; 488 nm), (2) green reflectance (GR; 515 nm), and (3) infrared reflectance (IR; 820 nm). Multicolor imaging compiles these three reflectance patterns into a single en face image, providing a pseudocolor representation of the fundus that simultaneously details retinal findings at varying depths. This allows a higher contrast compared with standard FC due to suppression of scatter light.

The three monochromatic images allow visualization of distinct information from specific layers of the retina and choroid. Blue laser is absorbed by macular pigment and can capture details of superficial retinal structures, whereas green laser is absorbed by hemoglobin and provides vascular details of the retina in addition to giving a good reflectance image of surface retinal disease. Because of the longer wavelength, near-infrared laser penetrates deeper into the retina, allowing better imaging of the retinal pigment epithelium and the choroid [32–34].

In presence of transparent media, green reflectance and blue reflectance images clearly show the increased reflection and wrinkling of the retinal surface associated with tractional ERM (Figure 5). Conversely, infrared reflectance reveals less detectability of epiretinal membranes. However, especially in elderly people, green-blue wavelengths may be blocked by lens opacities or the high reflectance from the surface of the retina may not allow an adequate visualization, thus resulting in images of poor quality. In these cases, infrared reflectance, with its deeper penetration, may be used. Using infrared imaging, Acquistapace et al. [35] identified three different categories of tangential traction associated with tractional epiretinal membranes in eyes with lamellar macular defects, categorizable as ERM foveoschisis according to the recent classification of Hubschman et al. [10]: (1) unidirectional, i.e., folds directing to a center of traction not in the fovea; (2) pluridirectional, i.e., more centers of traction with different directions of folds; and (3) concentric, i.e., all folds directing to the center of the fovea.

**Figure 11:**
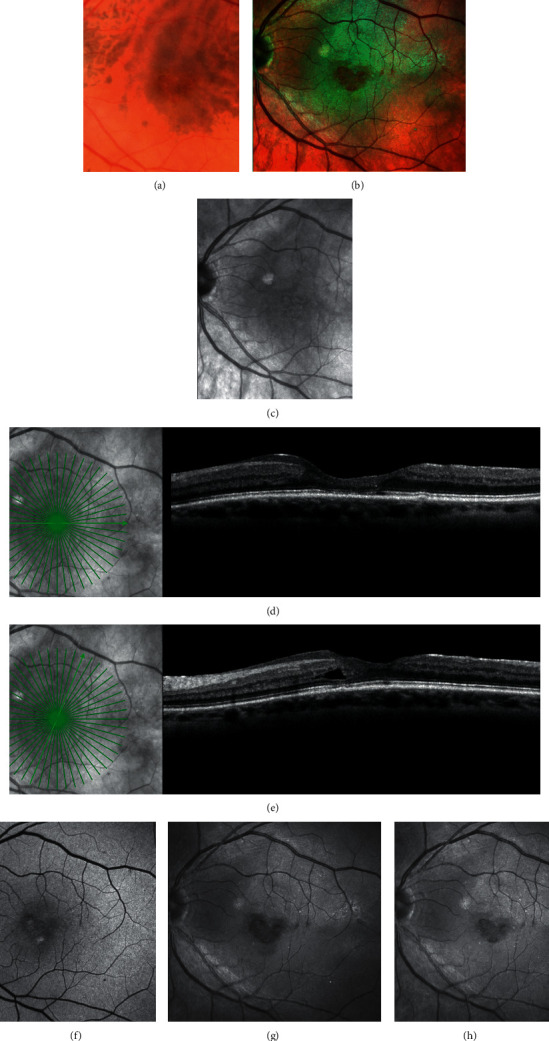
Multimodal imaging of a lamellar macular hole (LMH) with associated epiretinal proliferation (EP). On color fundus photograph (a), an oval reddish foveal lesion with no distortion or wrinkling of the surrounding retinal tissue is visible. On multicolor image (b), a yellowish area around the hole is visible, but its boundaries are not clearly delineated. On infrared reflectance image (c), no remarkable features are noted. On horizontal and oblique optical coherence tomography sections (d and e), EP in the form of material with medium reflectivity is observed on the retinal surface around the hole. On blue-fundus autofluorescence imaging (f), discrete areas of increased autofluorescent signal are visible. On blue-reflectance image (g), a sharply demarcated dark area, surrounding the hole, is evident. This area corresponds precisely to the surface covered by the EP on OCT scans. On green reflectance image (h), there are no peculiar findings corresponding to the area with EP.

Differently from conventional ERMs, EPs are typically not visible or detectable by ophthalmoscopy or color fundus photography [8, 9]. dell'Omo et al. have recently shown that BR and multicolor imaging enables the en face visualization of EP associated with LMH in the form of a sharply demarcated dark area and in the form of a yellowish area surrounding the hole, respectively [36] (Figure 11).

This has been related to the block of the blue light transmission caused by the yellow pigment contained within the EP tissue [37].

### 3.6. OCT Angiography

OCT angiography (OCTA) is a recently developed technique that provides depth resolved images of blood flow in the retina and choroid without injection of dye. With its capability of imaging the intermediate and deep retinal capillary plexuses, the OCTA opens a wealth of possibilities for disease description and quantification, including research into pathogenesis of disease.

In a retrospective study, Pierro et al. [38] analyzed 10 eyes with lamellar defects associated with tractional ERM but not with EP (thus comparable to ERM foveoschisis) and compared them with healthy controls. They found that the foveal avascular zone (FAZ) area in the superficial capillary plexus (SCP) was similar in the two groups whereas the FAZ area in the deep capillary plexus (DCP) was recognizable only in 30% of the cases with LMH. The eyes where the FAZ was not recognizable at the deep capillary plexus (DCP) presented a nonspecific, irregular cystic pattern. The vessel density at the superficial capillary plexus (SCP), DCP, and choriocapillaris did not differ between eyes with LMH and controls.

In a study by Ahn et al. [39], 19 eyes with LMH were studied with OCTA and compared with 19 age- and gender-matched normal controls. Axial length, subfoveal choroidal thickness, and vessel density (VD, i.e., the percentage of the area occupied by vessels in a selected region) of the choriocapillaris in the LMH group did not vary from normal controls. Since no specific details are provided in the paper, it is not possible to evaluate how many of the lesions considered by the authors could be classifiable as true LMH or ERM foveoschisis.

**Figure 12:**
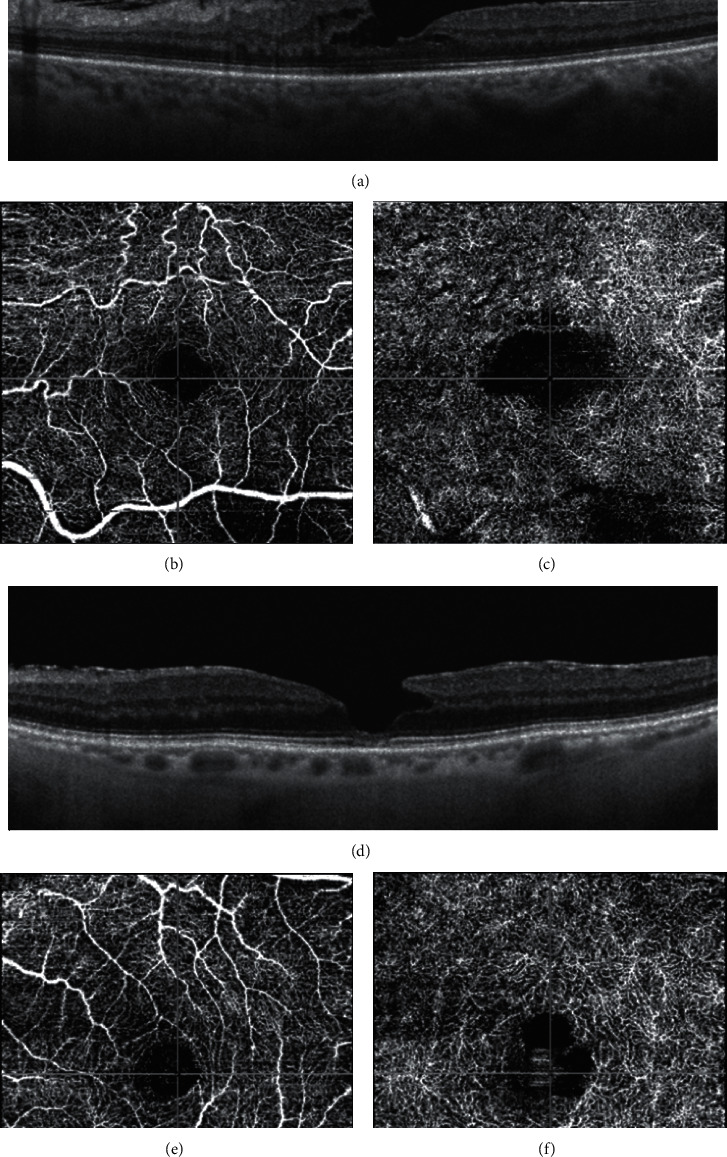
Optical coherence tomography (OCT) and OCT angiography of the eyes with epiretinal membrane (ERM) foveoschisis and lamellar macular hole (LMH). The tractional ERM associated with the foveoschisis (a) causes distortion and tortuosity of the superficial vessels (b). The foveal avascular zone (FAZ) of the deep capillary plexus appears enlarged (c). In the eye with LMH (d), the superficial vessels are not distorted (e). The FAZ of the deep capillary plexus appears enlarged with an irregular contour (f).

Yeo et al. [40] investigated the microvascular changes in 63 eyes with LMH (42 tractional and 21 degenerative according to the definition of Govetto et al. [15]) comparing the FAZ area and foveal and parafoveal VD with those obtained in a control group. Compared with control eyes, those with tractional LMH had smaller FAZ area, higher foveal VD, and lower parafoveal VD whereas eyes with degenerative LMH had lower parafoveal VD in both plexuses. In addition, foveal VDs in both plexuses and parafoveal VD in SCP were significantly correlated with BCVA in eyes with degenerative LMH **(**Figure 12).

### 3.7. Fluorescein Angiography

Ophthalmic fluorescein angiography is an important clinical procedure used to investigate and document the status of the retinal and choroidal vascular systems. To date, there are few data in the literature on the use of fluorescein angiography in LMH. Gass originally described and increased fluorescent signal due to a window defect corresponding to the lamellar hole [1]. In the paper by von Rükmann et al., 46 eyes FTMH and 5 eyes with pseudoholes were analyzed [41]. According to these authors, autofluorescence imaging with the cLSO makes the assessment of macular holes possible with accuracy comparable with that of fluorescein angiography [41].

In a recent report, Govetto et al. [23] performed macular narrow-angle (30 degrees) fluorescein angiography in a small series of 12 eyes diagnosed with ERM foveoschisis and found no apparent dye leakage in any of the included patients. Conversely, dell'Omo et al. found that eyes with LMH and eyes with ERM foveoschisis may show abnormal leakage at the posterior pole and in the periphery, focal vasculitis, and hyperfluorescence of the disc when studied with fluorescein angiography. These angiographic features may suggest the role of blood-retinal barrier breakdown and perhaps of inflammation in the pathogenesis of LMH [42].

## 4. Conclusions

Lamellar macular hole is a partial-thickness foveal defect, with variable morphologic features difficult to identify using biomicroscopy alone. Advancements in imaging and availability on the market of new equipment and processing techniques have deepened our knowledge about the morphologic characteristics, natural history, and long-term prognosis of this disease.

In the recent years, particular interest has been focused on the type of epiretinal membranes that can be associated with LMH, their relationship with histopathology studies, and their relevance from a pathogenetic point of view. The role of several imaging modalities has been explored for the detailed study of these membranes and other morphologic characteristics and new classification systems have been proposed. However, it is unknown at present which are the factors that may lead to a different pathway of evolution in the development of true LMH and ERM foveoschisis. Similarly, it is still not clear which imaging modality may best evidence the alleged loss of tissue that, according to most recent definitions, should be associated with true LMH.

Refinement in imaging techniques will hopefully provide in the near future further insights into this challenging vitreoretinal disease.

## Data Availability

The datasets generated during and/or analyzed during the current study are available from the corresponding author on reasonable request.
